# Grain Type Impacts Feed Intake, Milk Production and Body Temperature of Dairy Cows Exposed to an Acute Heat Event in Early Lactation

**DOI:** 10.3390/ani15071045

**Published:** 2025-04-04

**Authors:** S. Richard O. Williams, Matthew I. Knight, Tori C. Milner, Josie B. Garner, Peter J. Moate, Khageswor Giri, Murray C. Hannah, Joe L. Jacobs, William J. Wales, Leah C. Marett

**Affiliations:** 1 Agriculture Victoria Research, Ellinbank, VIC 3821, Australia; 2 Agriculture Victoria Research, Bundoora, VIC 3083, Australia; 3 School of Applied Systems Biology, La Trobe University, Melbourne, VIC 3086, Australia

**Keywords:** heat stress, cattle, feed type

## Abstract

The frequency, duration and intensity of heat events in Australia are forecast to increase. Different grain types result in different heat loads on animals, so grain selection could reduce the impact of heat exposure. Thirty-two multiparous Holstein cows at 86 days in milk were offered a basal forage diet plus one of four supplements: (1) BLY, rolled barley; (2) CAN, canola meal and rolled wheat; (3) CRN, disk-milled corn; or (4) WHT, rolled wheat. Cows were exposed to a 2-day heat wave in controlled-climate chambers. Overall, cows offered CAN had the lowest dry matter intake (DMI; 16.2 vs. 17.7 kg) but produced more energy-corrected milk (ECM; 34.9 vs. 29.6 kg) when compared with the other treatments. The results were similar during heat exposure. Cows fed CRN and CAN had the greatest body temperature (38.9 °C), and cows fed BLY had the lowest (38.4 °C). Despite this, cows fed BLY had the greatest reduction in DMI from the pre-challenge to the heat-challenge periods (−2.8 vs. −0.4 kg DM/d). There appears to be a small advantage to offering cows a concentrate with a greater protein concentration compared to one that has a greater concentration of fat or starch. The choice of grain to include in a dairy cow’s ration during summers with acute heat events may simply be an economic one.

## Simple Summary

The incidence and severity of hot weather events in Australia are expected to increase, and dairy cows are particularly susceptible to heat stress. Different grain types used in the diet of dairy cows may reduce the effect of hot weather on cow feed intake and milk production. Cows were offered one of four total mixed rations, each with the same amount of alfalfa hay, pasture silage and grain, but with a different grain in each ration. Measurements were made before, during and after a 2-day heat challenge. Overall, cows offered the diet with canola meal consumed the least feed but produced more milk compared with cows offered other diets. Also, cows offered the diet with barley had the lowest body temperatures. While there were few major differences between the diets, there appears to be a small advantage in offering cows the protein-rich canola meal over the more starch- or fat-rich grains tested. The choice of grain to include in a dairy cow’s ration during summers with acute heat events may simply be an economic one.

## 1. Introduction

In southeast Australia and similar temperate environments around the world, dairy production is predominantly a grazing-based system supplemented with pelleted concentrates or grain fed in the milking parlor [1]. In these environments, the changing climate has been accompanied by an increase in the duration and intensity of heat events [2,3].

Milk production can be negatively impacted by heat stress. Heat stress occurs when environmental conditions, such as air temperature, relative humidity, air movement and solar radiation, cause the animal’s body temperature to rise above their normal physiological range [4]. However, it is unclear whether the subsequent reduction in milk production is primarily associated with the cumulative effects of heat stress on the lactating dairy cow’s physiology and metabolism coupled with a reduction in dry matter intake (DMI), or a reduction in DMI alone [5]. Heat-stressed animals reduce their DMI as a survival strategy to reduce their metabolic heat load [6]. The metabolic heat load from feed is a combination of that generated during fermentation in the rumen, energy expenditure during digestive processes and the metabolism of digestion end products [7,8]. A reduction in DMI can also result in the slowing of the cow’s gut metabolism [9,10]. For short heat events (<4 days), the reduction in DMI accounts for most, if not all, of the reduction in milk yield [11,12], but this is not the case for longer heat events (7+ days) [5,13].

Metabolic heat load is influenced by a range of factors, including the characteristics of different feed ingredients, end products of digestion and energy partitioning between milk production and weight gain [14]. The fermentation of fibrous feeds in the rumen tends to result in a greater acetate-to-propionate ratio than the fermentation of grains [15,16]. This results in greater metabolic heat production since the metabolism of acetate generates more heat than the metabolism of propionate [10]. The differences between feed ingredients also extend to within grain types, with some fermenting quickly in the rumen (and creating heat), while others ferment more slowly and are predominantly digested in the small intestine, resulting in the release of glucose, which results in less metabolic heat than the metabolism of acetate [17,18].

The appetite of lactating dairy cows can be increased by feeding them diets with high protein concentrations such as canola meal, resulting in increased DMI and milk yield compared to those fed lower-protein diets [19]. However, the stimulatory effect on appetite has only been reported under non-heat-stress conditions. Given that the processing of feed in the rumen generates heat, increasing the appetite of dairy cows during hot weather could exacerbate the detrimental effects on lactating dairy cows including increased body temperature and energy expenditure [7,20]. One approach to ameliorate the impact of heat stress on lactating dairy cows is to feed them a low-fiber, high-energy grain diet. This approach may offset the decline in metabolizable energy intake associated with declining DMI by increasing the dietary energy concentration. Different grain options are available to feed dairy cows, providing options to manipulate starch, protein or fat concentrations. The aim of this approach is to reduce the heat increment during digestion, allowing the lactating dairy cow to improve its ability to thermoregulate its body temperature while maintaining DMI and milk production [21]. Garner et al. [12] showed that cows in late lactation that were fed corn grain in place of wheat grain and then exposed to a controlled 4-day heat challenge voluntarily consumed more forage than their wheat-fed counterparts. This difference in DMI resulted in a small increase in milk yield and lower respiration rates [12]. The impact that different grain types may have on DMI, milk production, and heat production during a single acute heat event in early lactation is unknown.

The objective of our experiment was to investigate the impact of different grain types (barley, canola meal plus wheat, corn, and wheat) in a low-fiber, high-energy, mixed-ration diet on the DMI, milk production and body temperature of lactating dairy cows exposed to a single heat event in a controlled-environment chamber. When cows are exposed to this single heat event, we hypothesized that the following would occur: (1) the DMI, milk yield and body temperature of cows would be greatest in those offered a diet containing canola meal compared to those fed the other diets; and (2) the DMI, milk yield and body temperature of cows fed a basal diet plus barley, corn or wheat grain would not differ.

## 2. Materials and Methods

The experiment was performed in accordance with the Australian Code of Practice for the Care and Use of Animals for Scientific Purposes [22] and approved by the DJPR Agricultural Research & Extension Animal Ethics Committee (AEC number: 2018-10, 16 October 2018). This experiment was conducted at Agriculture Victoria, Ellinbank SmartFarm, Victoria, Australia (38°14′ S, 145°56′ E), using controlled-climate chambers.

### 2.1. Animals and Diets

Thirty-two multiparous, lactating, Holstein-Friesian cows producing 35.1 ± 3.76 kg milk/d (mean ± standard deviation) with 3 ± 0.8 lactations, 578 ± 48.2 kg body weight, 86 ± 23.6 days in milk, 5.0 ± 0.9 years of age, 54 ± 58.7 balanced performance index (BPI; DataGene, Bundoora, Victoria, Australia; 0 = national breed mean) and 100 ± 3.1 heat tolerance breeding value (Htol_BV; DataGene, Bundoora, Victoria, Australia; 100 = national breed mean) were used in our experiment. Each cow was assigned to one of four mixed-ration diet treatments: (1) basal diet plus 8 kg DM rolled barley grain (BLY); (2) basal diet plus 2 kg DM solvent-extracted canola meal and 6 kg DM rolled wheat grain (CAN); (3) basal diet plus 8 kg DM disk-milled corn grain (CRN); or (4) basal diet plus 8 kg DM rolled wheat grain (WHT). The basal diet contained 5 kg DM alfalfa hay; 9 kg DM pasture silage (predominantly perennial ryegrass, *Lolium perenne* L.); 0.2 kg DM mineral mix (Ca 134 g/kg, Mg 110 g/kg, P 60 g/kg, Zn 6.4 g/kg, Mn 2.4 g/kg, Cu 1.2 g/kg, I 80 mg/kg, Co 100 mg/kg, Se 24 mg/kg, Vitamin A 165 IU/g, Vitamin D3 24 IU/g, Vitamin E 800 mg/kg); and 42 mL bloat drench (271 g/L alcohols, C12-15 ethoxylated; VicChem, Coolaroo, Victoria, Australia). The split between basal diet and grain was adjusted to ensure that each diet met the nutritional requirements of the cows [23]. All diets were offered as a total mixed ration and were not intended to be isoenergetic nor isonitrogenous.

### 2.2. Experimental Design and Heat-Challenge Schedule

The 4 treatment diets were organized in a 4-row-by-6-column design as shown in Table 1. Each row corresponds to a cohort, each column to a controlled-climate chamber and each cell to an individual cow. All 4 treatment diets occurred once in each controlled-climate chamber, and, within each cohort, each diet occurred in either one or two chambers. The pair of diets that occurred twice differed for each cohort to achieve near-balance and a total of 6 treatment replications over the 4 cohorts of the experiment. Treatment allocation was randomized according to the row–column design, by the permutation of rows and permutation of columns. Provision for the possibility of drop-outs due to animal health or behavior was made by preparing 8 cows per cohort, 2 on each diet treatment, rather than just the required 6 cows. This meant that a cow on a diet with a single replication in a cohort could be substituted with a cow on the same diet, or a cow on a doubly replicated diet treatment could be substituted with a cow in one of the singly replicated diets. In the latter case, this would alter the pattern of doubly replicated treatments for the cohort. Consequently, treatment selection in a subsequent cohort would be swapped to preserve equal replication and near-balance in the trial as much as possible. Accordingly, each of the 4 diet treatments was assigned to 8 cows, with 4 cohorts corresponding to 4 calving-date blocks, so that the treatment groups were similar in the average and standard deviation for cow body weight, 7-day milk yield, lactation number and balanced performance index and heat tolerance breeding values. This was achieved using a covariate design [24] implemented in GenStat 19 (VSN International Ltd., Hemel Hempstead, UK).

At the commencement of the experiment, a covariate period between days 1 and 4 enabled the measurement of milk yield, milk composition and cow body weight, with all cows offered 7 kg DM of wheat during milking and about 16 kg DM of pasture (predominantly perennial ryegrass) per day in a paddock.

**Figure 1:**
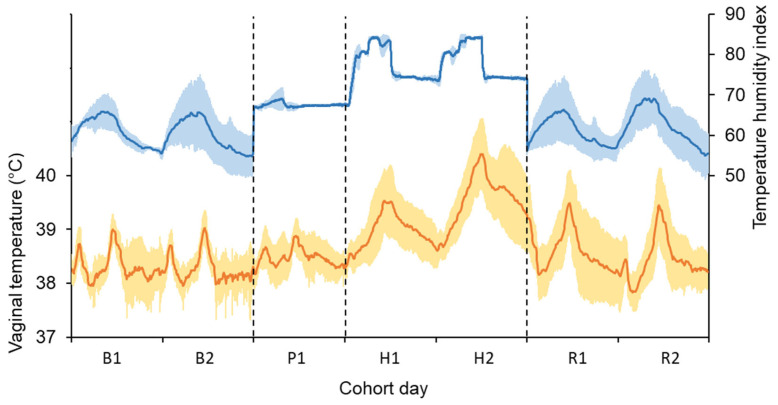
Mean environmental conditions experienced by the cows (blue line) and mean vaginal temperature of all cows (orange line) during the base (B), pre-challenge (P), heat-challenge (H) and recovery (R) period. Shading bands show ± one standard deviation ($\hat{\sigma }$) mapped relative to the mean ($\hat{\mu }$). The pre-challenge and heat challenge were conducted in controlled-climate chambers. Cows were kept in ambient conditions at all other times.

During the transition (days 5 to 7) and adaptation to diet (days 8 to 18), cows were fed their assigned mixed ration in individual feed stalls within a well-ventilated animal house [25], with water offered to all animals at least once during each feeding period. After feeding, cows were moved to a 560 m^2^ (over 20 m^2^ per cow), roofed loafing pad with rubber flooring (Kura Multiflex; Gummiwerk Kraiburg Elastik GmbH, Tittmoning, Germany) with ad libitum access to water. The final two days of the adaptation period were defined as the base period (days 17 to 18) for descriptive purposes only (i.e., in Figure 1).

In the pre-challenge period (day 19), cows were housed in individual controlled-climate chambers [26] in thermoneutral conditions (20 °C and 60% relative humidity, RH) with ad libitum access to water.

For the heat-challenge period (days 20 to 21), cows remained in their assigned controlled-climate chambers but were exposed to the following heat-challenge conditions with ad libitum access to water: from 06:00 h to 11:59 h, the set conditions were 30 °C and 50% RH (Temperature Humidity Index, THI = 80.1), from 12:00 h to 17:59 h, the setpoints were 33 °C and 50% RH (THI = 84.2), and from 18:00 h to 05:59 h, the setpoints were 25.5 °C and 60% RH (THI = 74.5). The THI was calculated using Equation (1) [27]:

THI = Tdb + (0.36 × Tdp) + 41.2(1)

where T_db_ = hourly dry bulb temperature (°C);

T_dp_ is the dew point temperature (°C);

T_dp_ = (237.3 × b)/(1.0 − b); b = [log(RH/100.0) + (17.27 × T_db_)/(237.3 + T_db_)]/17.27;

and RH = relative humidity (%).

During the recovery period (days 22 to 23), the lactating dairy cows were returned to the animal house and loafing pad in ambient environmental conditions.

### 2.3. Feeding Regime, Feed Intake and Feed Composition

Each diet was offered in two equal portions immediately following morning and afternoon milking. Individual animal refusals were collected and recorded after each feeding time.

Dry matter concentration was determined for representative individually collected samples of grains, canola meal and minerals that were collected over 2 consecutive days of each week of the experiment, while representative samples of alfalfa hay were collected every morning, and representative samples of pasture silage were collected at each feeding. Refused feed was collected, weighed and sampled immediately before the start of the next feeding period. Dry matter concentration was determined for all the representative samples of offered feed and refused feed by drying the samples in a forced-draft oven at 105 °C for 24 h.

Samples of the individual offered grains, canola meal, alfalfa hay and silage collected daily were stored at −18 °C until further processing. Samples were bulked by feed type within the period (pre-challenge, heat challenge and recovery) and cohort. Bulked samples were subsequently freeze-dried, ground to pass through a 0.5 mm screen (MEP rotor mill; Retsch GmbH, Haan, Germany) and then analyzed for crude protein (CP [28]; method 990.03), soluble protein (Cornell sodium borate–sodium phosphate buffer procedure), acid detergent fiber (ADF [28]; method 7.074), neutral detergent fiber (NDF [28]; method 2002.04), acid detergent lignin ([28]; method 949.04), non-fiber carbohydrate ([28]; method 992.09), starch ([28]; method 996.11), ash ([28]; method 942.05), crude fat (ether extract, EE [28]; method 2003.05), sodium, potassium, calcium, magnesium, phosphorous and sulfur (CEM Dairy One Digestion Method [29]) and chloride (Metrohm Application Bulletin No. 130/4 e). Total digestible nutrients (TDNs) were predicted by the model of Weiss et al. [30]. Metabolizable energy was calculated using Equation (2) [31].

ME (MJ/kg) = 14.55 − 0.0155 × ADF (g/kg)(2)

The composition of the main dietary ingredients is shown in Table S1. The nutrient composition of each of the individual ingredients was then used to determine the nutrient composition of each diet (Table 2).

### 2.4. Milk Production

Cows were milked twice daily, at approximately 06:00 h and 15:00 h. Throughout the experiment, milk yield was measured for each cow at each milking. When cows were not in the controlled-climate chambers, milk yields were recorded using a DeLaval Alpro milk metering system (MM25; DeLaval International, Tumba, Sweden), with milk samples for composition analysis collected during the covariate and recovery periods. When cows were in the controlled-climate chambers (pre-challenge and heat challenge), milk yield measurements were made by collecting and weighing the milk from individual cows. Milk samples for compositional analysis were collected during every milking. Fat, protein and lactose in milk samples were measured by means of a mid-infrared milk analyzer (Bentley FTS; Bentley Instruments, Chaska, MN, USA). Energy-corrected milk (ECM), standardized to 4.0% fat and 3.3% protein, was calculated using Equation (3) [32]:

(3) $$\mathrm{ECM}\;(\mathrm{kg}/\mathrm{cow}\;\mathrm{per}\;d)=\frac{\mathrm{milk}\;\mathrm{yield}\;(\mathrm{kg})\times (376\times \mathrm{fat}\%+209\times \mathrm{protein}\%+948)}{3138}$$

### 2.5. Physiology

The vaginal temperature of the cows was measured and recorded every 5 min throughout the experiment using iButton temperature data loggers (Maxim Integrated, San Jose, CA, USA) as described by Garner et al. [26], which were set to high-resolution mode. The duration of a vaginal body temperature greater than 38.8 °C was calculated as the number of minutes that vaginal temperature exceeded the 38.8 °C threshold. The area above 38.8 °C was calculated as the sum of the areas bounded by a horizontal line at 38.8 °C and the vaginal temperature-versus-time curve. The threshold of 38.8 °C was used as this was the mean vaginal temperature of cows during the thermoneutral period in the experiment of Garner et al. [26].

Cow rectal temperature was measured at approximately 06:00 h and 15:00 h on days 19 to 21 using a digital thermometer (Animal Thermometer AG-102 R01; AG-Medix LLC, Mukwonago, WI, USA). Rectal temperature was only used as part of animal welfare monitoring.

Cow respiration rates were assessed on cohort days 19 to 23. Respiration rates were measured by visually observing each cow at approximately 05:45 and 14:45 h. To measure a cow’s respiration rate, the number of breaths (flank movements) in a 20 s period was recorded and multiplied by three. The respiration rate was assessed twice per cow at each time point.

Panting scores were also assessed visually according to the criteria described by Gaughan et al. [33] at approximately 05:45 h, 12:00 h and 14:45 h on days 19 and 22 of the experiment.

Cow skin temperature was measured on cohort days 19 to 23 at approximately 06:00 h and 15:00 h. The surface temperature of the cow was measured using a non-contact infra-red thermometer (Oricom HFS1000; Oricom International, South Windsor, NSW, Australia) in object temperature mode, at the centroid of the left trapezius muscle.

### 2.6. Blood

Blood samples were collected from each cow at approximately 14:45 h via coccygeal venipuncture during the pre-challenge period (day 19), on day 2 of the heat-challenge period (day 21) and once during the recovery period (day 23). On each occasion, three 10 mL blood samples were collected: one sample into a vacutainer containing potassium EDTA for plasma collection and two samples into a vacutainer containing clotting activators for serum collection (BD Vacutainer System, Plymouth, UK).

With a syringe, approximately 0.1 mL of blood from the EDTA tube was removed immediately after collection and inserted into the reader chip of an auto-calibrated, portable blood gas analyzer (Epoc Host2 Zebra MC55A0, Epocal Inc., Ottawa, Ontario, Canada) as per the manufacturer’s instructions. The analytical variables determined were blood pH, partial pressure of CO_2_ (pCO_2_), partial pressure of O_2_ (pO_2_), Na^+^, Cl^−^, glucose, lactate, creatinine and hematocrit (Hct). Instrument-calculated values of bicarbonate (cHCO_3_^−^), total CO_2_ (cTCO_2_), hemoglobin (cHgb) and oxygen saturation (cSO_2_) were also recorded.

The remaining sample was then placed on ice before centrifugation within 30 min of collection at 1500× *g* and 4 °C for 10 min. Plasma was decanted into storage vials and stored at −20°C until analysis. The two samples for serum collection were kept at 25 °C for 1.5 h before centrifugation at 1300× *g* and 25 °C for 10 min. Serum was decanted into storage vials and stored at −20 °C until analysis. A subset of serum samples was transported on ice to Regional Laboratory Services (Benalla, Victoria, Australia) within 24 h of collection. Serum samples were analyzed for concentrations of beta-hydroxy butyrate (BHB), non-esterified fatty acids (NEFAs), urea total protein, albumin, glucose, haptoglobin and phosphorus using a Kone 20 XT clinical chemistry analyzer (Thermo Fisher Scientific, Waltham, MA, USA), with reagents supplied by Randox Laboratories (Crumlin, UK) for fatty acids and blood urea N (BUN) and Regional Laboratory Services (Benalla, Victoria, Australia) for BHB, albumin and total protein. Haptoglobin concentration was measured using a colorimetric rate assay [34].

### 2.7. Animal Welfare

Animal welfare was monitored using a heat stress risk rating [35]. Briefly, physiology observations (rectal temperature, respiration rate and panting score) were scored and the individual scores were summed to generate a heat stress risk total. Increased monitoring was initiated at a heat stress risk total of 1, and the heat challenge was ended early for cows that had a score greater than 2.

### 2.8. Statistical Analyses

Six cows were excluded from the analysis: one cow due to poor temperament within the controlled-environment chambers (1 CAN), one cow due to illness unrelated to the experiment (1 WHT), and four cows had their heat challenge ended early after exceeding the predetermined threshold for the heat stress risk total (1 BLY, 2 CAN, 1 WHT).

Only respiration rates and skin temperatures from the afternoon measurements were included in the statistical analysis as they were deemed to represent when the cows were most heat-stressed.

Daily data on milk yield, ECM yield, fat, protein and lactose concentration and amount in milk yield, DMI and intake of dietary nutritive components, mean, minimum and maximum vaginal temperatures, duration of vaginal temperature (minutes) above 38.8 °C, respiration rate, skin temperature and blood analytes were analyzed by linear mixed models that were fitted using restricted maximum likelihood (REML) in GenStat 21 (VSN International Ltd., Hemel Hempstead, UK), with the individual cow as the unit of analyses. The fitted mixed model can be written in the following equation form:

*y* = *μ* + *βy_cov_* + *c* + *k* + *t* + *p_t_* + *tp_t_* + *dp_t_* + *tdp_t_* + *A* + *KD* + *ε_AD_* (4)

where y is the response variable of interest, *µ* is the overall constant (grand mean), *y_cov_* is the mean value of the same variable (when available) from the covariate period for each cow and *β* is the linear effect (fixed effect) of this covariate. The lower-case Latin letters are fixed effects, upper-case Latin letters are random effects, assumed to be normally distributed, and *ε_AD_* is a normally distributed residual error. *c* is the main effect of chamber, *k* is the main effect of cohort, *t* is the main effect of diet treatment, *p_t_* is the main effect of treatment period, *tp_t_* is the interaction between diet treatment *t* and treatment period *p_t_*, *dp_t_* is the effect of day within a treatment period *p_t_*, and *tdp_t_* is the effect of day within a treatment within a treatment period. *A* is the effect of cow (animal) and *KD* is the effect of day within a cohort. The effect of cow within a day was used as a residual term *ε_AD_*. The chamber main effect (*c*) and cohort main effect (*k*) were included as fixed effects to ensure that the treatment means were directly adjusted for these factors. In contrast, cow (*A*), day within cohort (*KD*) and *ε_AD_* were used as random effects because they represented blocking factors that accounted for structured variability in the experiment. Histograms of residuals and plots of residuals vs. fitted values were examined for normality of distribution with constant variance. Body vaginal temperature (minutes) above 38.8 °C was logarithmically (base 10) transformed to satisfy these distributional assumptions.

Diet treatment means across the 5 days of measurement were estimated using the predicted main effects from the fitted model. The treatment means within each treatment period were provided by the interaction between diet treatment and treatment period from the model. Contrasts were formed and specific hypotheses tested by a *t*-test using the predicted means and their variance–covariances derived under the mixed model (Equation (4)). Changes due to heat stress were estimated as contrasts between the mean under 2 days of heat stress and the pre-challenge day. Duncan’s letters indicating significant differences amongst the comparisons of treatment means were based on Fisher’s unprotected Least Significant Difference (LSD) test. When Fisher’s unprotected LSD was used, the comparisons were tested even when the *p*-value for the term generating the means was not statistically significant. Fisher’s unprotected LSD is appropriate when there is a small number of comparisons, and these comparisons are used to address prior hypotheses [36].

## 3. Results

### 3.1. Conditions Experienced During the Experiment

Weather conditions during the base period (ambient conditions) were an air temperature of 14.8 ± 2.96 °C (daily mean ($\hat{\mu }$) ± standard deviation ($\hat{\sigma }$)), relative humidity of 73 ± 12.1% and THI of 60 ± 3.6. During the pre-challenge (in chambers) period, the cows experienced an air temperature of 19.0 ± 0.43 °C, relative humidity of 70 ± 3.9% and THI of 67 ± 0.5. During the heat-challenge (in chambers) period, the cows experienced an air temperature of 26.6 ± 3.57 °C, relative humidity of 62 ± 5.6% and THI of 77 ± 4.4. The weather conditions during the recovery period (ambient conditions) were an air temperature of 15.7 ± 3.18 °C, relative humidity of 72 ± 11.3% and THI of 62 ± 3.9. The daily pattern in the THI experienced by the cows during the base, pre-challenge, heat-challenge and recovery periods is shown in Figure 1.

The coefficient of variation in the THI was 0.01 during the pre-challenge period, 0.06 during the heat challenge and 0.06 during the recovery period.

### 3.2. Dry Matter Intake

**Figure 2:**
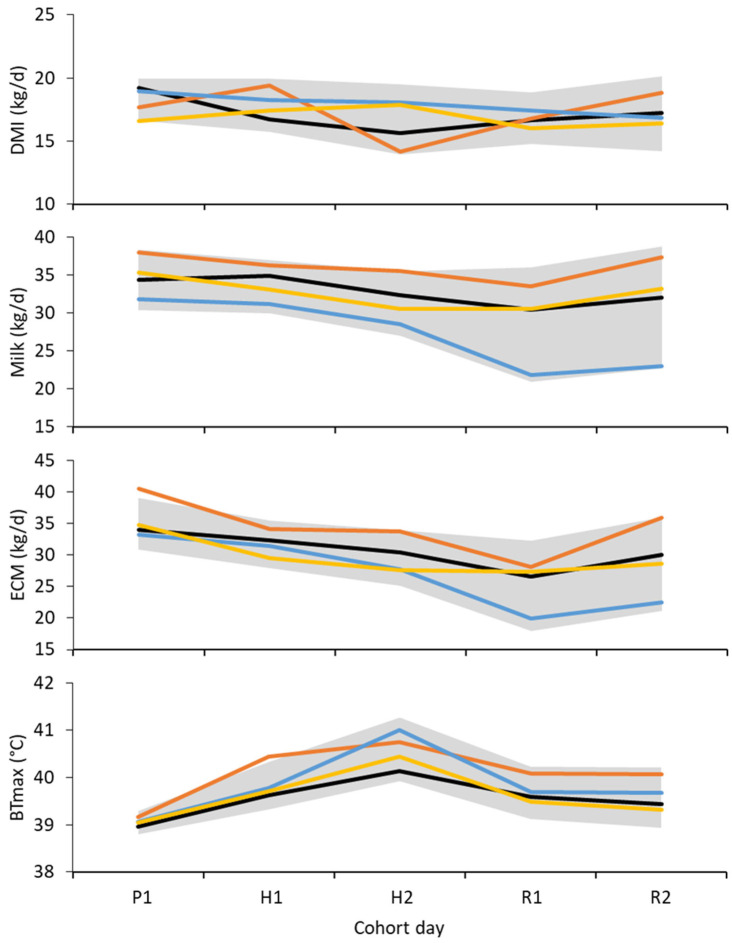
Mean daily dry matter intake (DMI), milk yield (Milk), energy-corrected milk yield (ECM) and maximum vaginal temperature (BTmax) of cows offered barley (black line), solvent-extracted canola meal plus wheat (orange line), corn grain (blue line) or wheat grain (gold line) during the pre-challenge (P), heat-challenge (H) and recovery period (R). The gray band is ± one standard deviation ($\hat{\sigma }$) mapped relative to the mean ($\hat{\mu }$). The pre-challenge and heat challenge were conducted in controlled-climate chambers. Cows were kept in ambient conditions at other times.

Across the 5 days of measurement (cohort days 19 to 23, inclusive), the average DMI and metabolizable energy intake (MEI) were both lowest in cows offered the CAN diet and greatest in those offered the BLY and CRN diets (Figure 2 and Table 3). The intake of crude protein was greatest in cows offered the CAN diet, despite them having the lowest DMI. The intake of both starch and fat was greatest in cows offered the CRN diet, and the intake of starch was lowest in those offered the CAN diet.

During the pre-challenge period, DMI was lowest in cows offered the WHT and CAN diets and greatest in those offered the BLY diet (Table 3). The intake of ME was lowest in those cows offered the CAN and WHT diets. Compared to cows offered WHT, the intake of ME in cows offered BLY was greater. The intake of both starch and fat was greater in cows offered the CRN diet.

During the heat challenge, DMI was lowest in those cows offered CAN and greatest in those offered CRN and WHT (Table 3). Compared to the WHT diet, DMI and MEI was lower in cows offered the CAN diet but not in those offered BLY or CRN. The intake of starch was greater in cows offered CRN and WHT compared to other diets, while the intake of fat was greater in cows offered CRN compared to the other diets.

During the recovery period, there was no effect of diet on DMI or MEI (Table 3). The intake of starch was greatest in cows offered the CRN diet. Fat intake was greatest in cows offered CRN.

From the pre-challenge period to the heat challenge, DMI, MEI and protein, NDF, starch and fat intake declined for cows offered the BLY, CAN and CRN diets, but increased for cows offered WHT (Table 3).

### 3.3. Milk Yield

Over the 5 days of measurement (cohort days 19 to 23, inclusive), the average milk yield ranged from 31.4 kg/d (CRN cows) to 35.2 kg/d (CAN cows). Compared with all other treatments, ECM yield was greater for cows offered the CAN diet (Table 4). Similarly, fat and protein yield were greater in cows offered the CAN treatment compared with all other treatments. The concentration of fat in the milk was unaffected by treatment, but the concentration of protein was lowest in milk from cows offered the WHT diet and greatest in milk from cows offered the BLY diet.

During the pre-challenge period, there was no difference in milk yield between treatments. The mean ECM yield from cows offered the BLY, CRN and WHT diets (32.6, 33.6 and 34.5 kg/d, respectively) was less than that from cows offered the CAN diet (1.3 kg/d) (Table 4). The yield of milk fat was greater from cows offered CAN compared to the other diets, with no difference between the BLY, CRN and WHT diets. Protein yield was lowest from the cows offered the CRN and BLY diets, and greatest from the cows offered the CAN and WHT diets.

During the heat challenge, diet had no effect on milk yield. The yield of ECM, fat and protein from the CAN cows was greater than that of ECM from the WHT cows, with the BLY and CRN diets having intermediate effects (Table 4).

During the recovery period, milk yield was greater from cows offered the CAN diet compared to those offered BLY and CRN, but not WHT. There was no difference in milk yield between cows offered the BLY, CRN or WHT diets (Table 4). The yield of ECM was affected by diet, with the ECM yield of cows offered the CAN diet being greater than that of cows offered the BLY or CRN diets. The CAN cows also had greater fat and protein yields than the CRN cows.

From the pre-challenge period to the heat challenge, there was a decline in all parameters, but there was only an effect of diet on the change in protein concentration in milk, where cows offered the BLY and CRN diets had a smaller decrease than the CAN cows.

### 3.4. Physiology

Over the 5 days of measurement (cohort days 19 to 23, inclusive), the mean vaginal temperature was affected by diet (Table 5). The mean vaginal temperature was lowest in cows offered the BLY diet, intermediate in those offered the WHT diet and greatest in cows offered the CAN or CRN diets. Similar effects of diet were seen for the maximum vaginal temperature and the duration for which vaginal temperature was greater than 38.8 °C. The respiration rate was unaffected by diet. The skin temperature of the cows offered the BLY diet was lower than that of the cows offered the CAN or CRN diets.

During the pre-challenge period, the mean vaginal temperature was not affected by diet. The minimum vaginal temperature was lower in the BLY and WHT cows than in the CRN cows. There was no effect of diet on the maximum vaginal temperature or the duration for which vaginal temperature was greater than 38.8 °C. Diet had no effect on the respiration rate. The skin temperature of the BLY cows was lower than that of the CAN and CRN cows.

During the heat challenge, all vaginal temperature parameters were affected by diet (Table 5). The mean, minimum and maximum vaginal temperature and the duration for which vaginal temperature was above 38.3 °C were lower in cows offered the BLY diet than cows offered the CRN diet. There was no effect of diet on the respiration rate or skin temperature.

During the recovery period, the mean vaginal temperature was affected by diet, ranging from 38.0 °C (BLY cows) to 38.7 °C (CAN cows) (Table 5). The minimum and maximum vaginal temperature and the duration for which vaginal temperature was greater than 38.8 °C were also affected by diet, being lower in the BLY and WHT cows than in the CAN cows. The respiration rate was not affected by diet. The skin temperature of the BLY cows was lower than that of the CAN cows.

From the pre-challenge period to the heat challenge, there was no effect of diet on the change in the mean, minimum or maximum vaginal temperature, or the change in the duration for which vaginal temperature was greater than 38.8 °C.

### 3.5. Blood

During the pre-challenge period, the only blood parameters (Table 6) affected by diet were the concentrations of haptoglobin and sodium. Haptoglobin was greater in the BLY cows than in the CRN cows, and sodium was lower in the CRN cows than in the WHT cows.

During the heat-challenge period, the concentration of NEFA was greatest in cows offered the CAN diet, intermediate in those offered the CRN and WHT diets and lowest in those offered the BLY diet. There was no effect of diet on the concentrations of BHB, glucose, haptoglobin or sodium.

During the recovery period, there was no effect of diet on any of the blood parameters measured.

From the pre-challenge period to the heat challenge, the change in the concentration of NEFA was positive for cows on all diets, but the change in cows offered the CAN diet was greater than the change in cows on the other diets.

## 4. Discussion

### 4.1. Canola

Cows offered the CAN diet ate less feed and produced more ECM, but body temperatures were not different from cows offered the other diets over the 5 days of measurement. Thus, we reject the DMI and body temperature portions of our first hypothesis, but the results support the milk yield portion of our hypothesis, despite cows offered canola meal having the lowest DMI and MEI. This result was consistent over the full 5 days of measurement and during the 2-day heat-challenge period.

The reason that the cows offered the CAN diet ate less is not clear. Greater metabolic heat from protein digestion or the excretion of excess nitrogen [37] than other diet components is a possibility, but we have no evidence to support this. A change in fermentation induced by the high protein concentration in the CAN diet may have occurred, but we did not find any previous reports linking a change in the ratio of volatile fatty acids to DMI, so we consider this scenario unlikely. We were not able to find any previous reports on the effect of feeding high-protein diets to ruminants experiencing acute heat stress. This is an area of research that warrants further investigation.

There appears to be a small advantage in the efficiency of milk production in cows offered a diet with a greater protein concentration compared to one that has a greater concentration of fat or starch. For example, despite a difference in ME intake (24 MJ, equivalent to ~5 L of milk [31]) in favor of the WHT cows during the heat challenge, the ECM yield was greater in the CAN cows than in the WHT cows. Our results are similar to those reported previously under ambient weather conditions. Cows fed a diet with the same ME as the control diet but with a greater protein intake, supplied through the addition of canola meal, produced more milk [19]. This extra milk yield was thought to be supported by the greater intake of pasture than the control cows, with possible influences of a more stable ruminal pH and a greater supply of more balanced amino acids [19]. Our treatment diets were not analyzed for amino acid profiles, nor were measurements of ruminal pH collected; therefore, the mechanisms of intake control linked to the protein intake of cows remain speculative. However, the milk advantage of our CAN diet over the other diets appears unrelated to heat exposure and is more likely influenced by the nutritional composition of the diet and overall cow metabolic factors.

The milk advantage of the CAN diet over the other diets during the heat challenge could have come at the cost of body reserves, as indicated by the greater concentrations of NEFA in the blood of the CAN cows than the cows offered the other diets during our 2-day heat challenge (Table 6). This mobilization of body reserves during our short-term heat challenge is similar to the findings in the report of Garner et al. [12], who used a short 4-day heat challenge. However, our finding is different from those in reports of longer periods of heat stress (greater than 5 days), where body reserves were not mobilized (e.g., [5]). Alternatively, differences in energy expenditure to maintain homeostasis might explain the CAN cows producing more ECM than the other cows with less feed. The CAN diet did not bestow any advantage on the physiological indicators of heat stress—respiration rate, skin temperature or vaginal temperature—during the heat-challenge, indicating that cows in all treatments experienced a similar degree of heat stress. This suggests that the reason the cows offered the CAN diet produced more ECM from less feed during the heat challenge than the other cows is likely a greater mobilization of body reserves.

### 4.2. Barley, Corn and Wheat

Dry matter intake and milk yield over the 5 days of measurement were not different between the cows fed the BLY, CRN and WHT diets. However, the cows offered the BLY diet had a lower body temperature than the CRN cows. Thus, we accept the DMI and milk portions of our second hypothesis, but reject the body temperature portion. However, differences in DMI and milk were not consistent across the periods of the experiment.

During the pre-challenge period, our BLY cows had greater DMI than our cows offered the WHT diet. Cows fed the WHT, BLY or CRN diets showed no difference in milk yield, but cows offered BLY had a lower yield of milk protein than the WHT cows. The greater DMI of our BLY cows compared to those offered WHT is in contrast to the no-difference result obtained when Friesian cows in ambient conditions were offered barley or wheat in a TMR [38]. However, our milk yield results are in agreement with previous work in Friesian cows offered a TMR [38] and Jersey cows offered grain prior to milking and forage after milking [39]. When the BLY and CRN diets were compared, there was no difference in DMI or milk yield, similar to other reports in which Friesian cows were fed either barley- or corn-grain diets in a TMR [38,40,41,42], or Jersey cows were offered grain before milking and forage after milking [39]. Again, when the CRN and WHT cows were compared, there was no difference in DMI or milk yield, but previous reports on the effect of corn versus wheat grain are equivocal. When dairy cows were fed corn grain followed by alfalfa hay, they ate more than cows fed wheat grain and then alfalfa hay [43], but another experiment similar to ours reported no difference in intake [12]. A similar story exists for milk yield, with our CRN and WHT cows showing no differences in milk but our CRN cows having a lower protein yield than our WHT cows. In the experiment of Moate et al. [43], cows offered a diet containing corn grain had greater ECM, greater fat yield and greater fat concentration than cows offered a diet containing wheat grain. This was also reported in the experiment of Garner et al. [12]. An explanation for these varied results in the literature could be the stage of lactation. The current experiment used cows at approximately 86 DIM. The work of Moate et al. [43] used cows at 171 DIM, and Garner et al. [12] used cows at 220 DIM. Milk fat concentration has been negatively correlated with the amount of starch digested post-ruminally [44]. This previous result [44] suggests that the greater starch digested post-ruminally by our CRN cows than our WHT cows should have resulted in a lower milk fat concentration in our CRN cows than our WHT cows. This was not the case in our cows nor in the cows of Moate et al. [43], who attributed the reduced milk fat in their wheat-fed cows to the presence of C18:1 trans-10, which was not measured in our work. Both our work and that of Reynolds et al. [44] were undertaken using cows in early lactation, but our diets consisted of different forms of starch, while Reynolds et al. [44] infused additional starch post-ruminally. The differences between our experiment and that of Reynolds et al. [44] mean that we favor the idea of sub-acute ruminal acidosis in our WHT cows as the explanation for the numerical differences observed in milk fat concentration in our work. A low pH was reported in the rumen of wheat-fed cows in the work of Moate et al. [43], supporting our speculation that sub-acute ruminal acidosis in our WHT cows overrode any effect of the site of starch digestion while cows were managed under ambient conditions.

During the heat challenge, our cows offered the BLY and WHT diets showed no difference in DMI, milk or milk composition. This supports our second hypothesis and could be due to the grains having similar nutritional profiles and minor differences in degradation characteristics [45,46]. Our BLY and CRN cows also showed no difference in DMI, milk yield or milk composition. This is perhaps less expected during heat challenge since barley and corn grain have different rates and extents of degradation in the rumen [46]. Corn is generally thought to degrade slowly and incompletely in the rumen and so should correspond to a lower rumen temperature than barley. A lower rumen temperature should have meant that the CRN cows could eat more than the BLY cows since rumen temperature affects intake [47,48], but this was not the case in our cows. Grain processing affects starch degradability, with greater processing resulting in greater degradability [45]. Given that our barley was simply rolled and our corn was disk-milled, it is possible that the more extensive processing of the corn grain resulted in the ruminal degradability of the two grains being similar. This would explain the lack of difference we observed in DMI and milk yield. However, if the barley and corn did have the same degradation characteristics post processing, the reason for the BLY cows having a lower body temperature than the CRN cows is not known. Previous reports comparing the feeding of barley to corn or wheat during a heat challenge have not been found, indicating that further work will be necessary if the merits of feeding barley during hot weather are to be more fully understood.

Our CRN and WHT cows showed no difference in DMI and milk yield during the heat challenge, but the CRN cows had a greater milk fat concentration than the WHT cows. This is similar to the results reported in a study where dairy cows were fed corn or wheat in a TMR during a sub-tropical summer [21]. A different result was reported when cows were fed corn or wheat grain prior to their forage [12]. Cows fed corn grain and then forage ate more forage, and hence more total DM, than cows fed wheat grain and then forage [12]. Neither the stage of lactation nor body temperature explains the differences between the results of Gonzales-Rivas et al. [21] and Garner et al. [12], since the cows in both studies were at a similar stage of lactation and both reported no difference in body temperature parameters. Cows offered slowly fermenting grains (such as corn) should produce more milk than cows fed fast-fermenting grains since more glucose (required for lactose production) is absorbed in the small intestine [21], but this was not the case in our experiment nor the experiment of Garner et al. [12]. It is possible that the extensive processing of our corn meant that the ruminal degradation characteristics of the corn and wheat were closer than intended, with both the disk-milled corn and the rolled wheat having similar effects on the cows during the heat challenge. This is reflected in the lack of a difference in the vaginal and skin temperatures of the CRN and WHT cows. Alternatively, the short duration of the heat-exposure period in our experiment may have been insufficient to result in consistent changes that were large enough to detect in our small group of cows.

### 4.3. Dietary Heat

Internal heat production is affected by diet composition. Slowly fermenting grains such as corn are expected to impose a lower heat load on animals than fast-fermenting grains such as wheat [21,49,50]. Regardless of the stage of lactation, the slower rate of fermentation of barley and corn compared to wheat [51] should have resulted in lower ruminal temperature in our cows offered the BLY and CRN diets compared to those offered the WHT diet. This is relevant because a greater ruminal temperature has been shown to reduce DMI [47,48]. Adding to the heat of fermentation is the heat of metabolism. Corn grain ferments more slowly in the rumen than wheat and so has a lower heat of fermentation, but corn grain generates more acetate, which has a high heat of metabolism [10], per unit of feed than wheat grain. However, the corn that escapes fermentation in the rumen results in the production of glucose, which has a low heat of metabolism [17,18]. While wheat ferments more rapidly in the rumen (high heat) the result is a low yield of acetate and thus a low heat of metabolism. It may be the case that the total heat production from corn- and cereal-grain-based diets is similar. This is consistent with our results and supported by the fact that there was no difference in the vaginal temperature, respiration rate or skin temperature between our cows offered the CRN diet and those offered the WHT diet. Ruminal pH could explain the differences observed in our work during the pre-challenge period since cows offered corn have been shown to have a greater minimum ruminal pH than cows offered wheat [43] and low pH has been shown to reduce intake [52]. However, without concurrent measurements of these possible explanatory parameters, these explanations remain speculative.

### 4.4. Limitations

Although our heat challenge was only of a 2-day duration, our cows did experience heat stress. This was illustrated by the declines in the DMI and milk yield and increases in the body temperature and respiration rate of our cows. Inducing heat stress with short periods of heat challenge has been reported previously [11,13,35]. Our short heat challenge is typical of heat events in southern Australia. However, it may not have been long or hot enough to fully induce the effects of the different treatments, highlighting that the duration of a heat event is an important factor requiring further investigation. Exposure to longer heat events has been shown to result in greater impact than shorter heat events [13], with DMI and milk yield decreasing with the length of the heat challenge [26]. While our results may apply to locations that experience short-term heat waves, they may not represent the effects that could be observed over a full summer or in a region that experiences long periods of continuously hot weather.

The number of cows participating in our experiment was relatively low, particularly for the WHT diet, where only four cows participated in the experiment. Combined with the between-cow variation observed, the low number of cows within treatments meant that some parameters with large numerical differences were not statistically different. Examples can be seen in the blood results, where there are two-fold differences between treatment means in plasma NEFA concentrations but no statistical difference.

The concentrates tested represented a relatively small proportion (36%) of the diets offered. Forage type has been shown previously to influence milk yield and ruminal responses to diet [35,53], so it is possible that the forage component of the diets was buffering the effect of the concentrate component.

## 5. Conclusions

Changing the type of grain included in the diet of dairy cows caused only minor changes to DMI or milk production when cows were exposed to a short-term controlled heat challenge. During the 5-day experiment, overall, the inclusion of canola meal in place of some of the wheat grain in the diet resulted in similar DMI and increased milk yield. However, during the 2-day exposure to heat, cows offered canola meal ate less and produced more ECM than cows offered wheat. Therefore, in our short-term experiment, it appears that there is an advantage to feeding cows a concentrate supplement with a greater crude protein concentration.

Dry matter intake over the 5-day experiment was not improved when cows were offered barley or corn grain in place of wheat, nor was there any advantage in milk production. However, cows offered barley generally had lower body temperatures than cows offered corn or wheat, which may have implications when cows are exposed to heat events of longer durations.

Overall, the only advantages of any one diet over any of the other diets tested was in ECM yield (diet with canola meal) and body temperature (diet with barley grain), but further investigation is warranted due to the low numbers of cows in our experiment. However, the choice of grain to include in a dairy cow’s ration during acute heat events may simply be driven by economics.

## Figures and Tables

**Table 1:** Blocking structure and treatment allocation to chambers for each of the 4 cohorts.

	Chamber 1	Chamber 2	Chamber 3	Chamber 4	Chamber 5	Chamber 6
Cohort 1	WHT	BLY	CAN	WHT	CRN	CAN
Cohort 2	CRN	CAN	BLY	BLY	WHT	WHT
Cohort 3	CAN	WHT	CRN	CRN	BLY	BLY
Cohort 4	BLY	CRN	WHT	CAN	CAN	CRN

**Table 2:** Nutrient and mineral composition of the four treatment diets (g/kg DM unless otherwise stated) fed to lactating dairy cows during the experiment.

	BLY ^1^	CAN	CRN	WHT
Crude protein	154	184	144	159
Soluble protein (% CP)	39.2	41.0	39.7	41.8
Acid detergent fiber	236	241	226	227
Neutral detergent fiber	354	352	326	337
Acid detergent lignin	45.8	49.2	41.9	43.6
Non-fiber carbohydrate	392	364	428	410
Starch	211	184	263	237
Crude fat	28.8	28.3	35.4	27.4
Ash	72.6	72.9	66.6	68.0
Total digestible nutrients	672	669	698	683
Calcium	5.4	6.0	5.2	5.4
Magnesium	2.0	2.4	2.0	2.0
Sodium	2.3	2.3	2.3	2.3
Potassium	20.1	20.6	19.7	19.7
Chloride	7.8	7.6	7.5	7.6
DCAD ^2^ (meq./100 g DM)	24.0	22.1	24.1	23.0
Copper (mg/kg DM)	7.6	8.7	7.2	8.8
Sulfur	2.5	3.1	2.4	2.6
Metabolizable energy (ME, MJ/kg DM)	11.3	11.4	11.5	11.5

^1^ BLY = basal diet plus barley grain; CAN = basal diet plus canola meal and wheat grain; CRN = basal diet plus corn grain; WHT = basal diet plus wheat grain. ^2^ DCAD = dietary cation–anion difference.

**Table 3:** Mean dry matter and macronutrient intake (kg DM/d unless specified otherwise) during the periods of the experiment and changes between periods.

	BLY ^1^	CAN	CRN	WHT	SED
*Animals (n)*	5	3	6	4	
*5-day mean*					
Dry Matter Intake	18.2 ^b^	16.2 ^a^	17.9 ^b^	17.0 ^ab^	0.701
Crude Protein	2.02 ^ab^	2.26 ^b^	1.83 ^a^	1.95 ^a^	0.134
Neutral Detergent Fiber	2.97 ^ab^	3.27 ^ab^	3.32 ^b^	2.55 ^a^	0.409
Non-Fiber Carbohydrate	8.13 ^c^	3.99 ^a^	5.69 ^b^	7.05 ^c^	0.711
Starch	3.99 ^b^	3.26 ^a^	5.02 ^d^	4.51 ^c^	0.186
Fat	0.24 ^a^	0.25 ^a^	0.41 ^b^	0.26 ^a^	0.046
Metabolizable Energy (MJ/d)	166 ^b^	147 ^a^	165 ^b^	158 ^ab^	6.396
*Pre-challenge*					
Dry Matter Intake	20.5 ^b^	16.9 ^a^	18.8 ^ab^	16.6 ^a^	1.25
Crude Protein	2.34 ^ab^	2.46 ^b^	2.02 ^a^	1.94 ^a^	0.215
Neutral Detergent Fiber	3.68 ^b^	3.62 ^ab^	3.80 ^b^	2.74 ^a^	0.537
Non-Fiber Carbohydrate	8.91 ^c^	4.38 ^a^	5.87 ^c^	6.72 ^b^	0.830
Starch	4.28 ^b^	3.34 ^a^	4.91 ^c^	4.21 ^b^	0.292
Fat	0.29 ^a^	0.28 ^a^	0.43 ^b^	0.26 ^a^	0.056
Metabolizable Energy (MJ/d)	184 ^b^	154 ^a^	174 ^ab^	155 ^a^	11.4
*Heat challenge*					
Dry Matter Intake	17.3 ^ab^	15.4 ^a^	18.0 ^b^	17.7 ^b^	0.931
Crude Protein	1.89	2.10	1.86	2.05	0.167
Neutral Detergent Fiber	2.80	3.14	3.42	2.88	0.457
Non-Fiber Carbohydrate	7.66 ^c^	3.53 ^a^	5.68 ^b^	7.17 ^c^	0.753
Starch	3.66 ^b^	2.96 ^a^	4.93 ^d^	4.47 ^c^	0.229
Fat	0.21 ^a^	0.23 ^a^	0.40 ^b^	0.27 ^a^	0.049
Metabolizable Energy (MJ/d)	158 ^ab^	141 ^a^	167 ^b^	164 ^b^	8.51
*Recovery*					
Dry Matter Intake	18.1	16.7	17.3	16.6	0.929
Crude Protein	1.99 ^ab^	2.33 ^b^	1.70 ^a^	1.86 ^a^	0.161
Neutral Detergent Fiber	2.80	3.14	3.42	2.88	0.443
Non-Fiber Carbohydrate	8.21 ^c^	4.26 ^a^	5.62 ^b^	7.09 ^c^	0.732
Starch	4.16 ^b^	3.52 ^a^	5.16 ^d^	4.70 ^c^	0.237
Fat	0.25 ^a^	0.26 ^a^	0.40 ^b^	0.24 ^a^	0.0473
Metabolizable Energy (MJ/d)	165	151	159	154	8.512
*Pre-challenge to heat * ^2^					
Δ Dry Matter Intake	−2.8 ^a^	−1.5 ^ab^	−0.8 ^ab^	1.1 ^b^	1.190
Δ Crude Protein	−0.45 ^a^	−0.36 ^ab^	−0.16 ^ab^	0.11 ^b^	0.189
Δ Neutral Detergent Fiber	−0.87 ^a^	−0.48 ^ab^	−0.38 ^ab^	0.14 ^b^	0.395
Δ Non-Fiber Carbohydrate	−1.25 ^a^	−0.84 ^ab^	−0.19 ^bc^	0.45 ^c^	0.512
Δ Starch	−0.62 ^a^	−0.38 ^ab^	0.02 ^b^	0.26 ^b^	0.297
Δ Fat	−0.08 ^a^	−0.05 ^ab^	−0.03 ^ab^	0.01 ^b^	0.037
Δ Metabolizable Energy (MJ/d)	−26 ^a^	−13 ^ab^	−6 ^ab^	9 ^b^	10.8

**^1^** BLY = basal diet plus barley grain; CAN = basal diet plus canola meal and wheat grain; CRN = basal diet plus corn grain; WHT = basal diet plus wheat grain. ^2^ Δ variable = (heat-challenge variable − pre-challenge variable). ^a–d^ Means in the same row followed by different superscripts differ significantly (*p <* 0.05).

**Table 4:** Milk yield (kg/d) and composition (g/kg) during the periods of the experiment and changes between periods.

	BLY ^1^	CAN	CRN	WHT	SED
*Animals (n)*	5	3	6	4	
*5-day mean*					
Milk yield	31.7 ^ab^	35.2 ^b^	31.4 ^a^	32.7 ^ab^	1.52
ECM ^2^ yield	29.5 ^a^	34.9 ^b^	29.2 ^a^	30.2 ^a^	1.58
Fat yield	1.16 ^a^	1.44 ^b^	1.21 ^a^	1.18 ^a^	0.083
Protein yield	0.88 ^a^	1.06 ^b^	0.83 ^a^	0.91 ^a^	0.053
Fat concentration	41.0	37.2	40.7	35.7	6.34
Protein concentration	29.8 ^b^	27.6 ^ab^	28.8 ^ab^	27.5 ^a^	1.33
*Pre-challenge*					
Milk yield	33.3	37.0	33.7	35.6	1.90
ECM yield	32.6 ^a^	41.3 ^b^	33.6 ^a^	34.5 ^a^	2.22
Fat yield	1.32 ^a^	1.81 ^b^	1.48 ^a^	1.47 ^a^	0.135
Protein yield	1.01 ^a^	1.22 ^b^	0.92 ^a^	1.05 ^b^	0.076
Fat concentration	43.4	45.1	46.2	41.0	7.17
Protein concentration	31.4	31.2	29.2	29.1	1.50
*Heat challenge*					
Milk yield	32.5	34.9	32.5	32.0	1.69
ECM yield	30.1 ^ab^	34.5 ^b^	30.7 ^ab^	29.2 ^a^	1.84
Fat yield	1.18 ^ab^	1.42 ^b^	1.30 ^ab^	1.13 ^a^	0.107
Protein yield	0.92 ^ab^	1.04 ^b^	0.88 ^a^	0.88 ^a^	0.064
Fat concentration	40.1 ^ab^	36.7 ^ab^	41.8 ^b^	35.2 ^a^	6.86
Protein concentration	29.7	27.1	29.0	27.2	1.39
*Recovery*					
Milk yield	30.2 ^a^	34.5 ^b^	29.1 ^a^	31.9 ^ab^	1.70
ECM yield	27.2 ^a^	32.2 ^b^	25.5 ^a^	28.5 ^ab^	1.87
Fat yield	1.06 ^ab^	1.28 ^b^	0.98 ^a^	1.08 ^ab^	0.108
Protein yield	0.78 ^ab^	0.98 ^c^	0.72 ^a^	0.87 ^bc^	0.064
Fat concentration	40.6	33.7	36.7	33.5	6.88
Protein concentration	29.1	26.2	28.3	27.0	1.39
*Before challenge with heat * ^3^					
Δ Milk yield	−0.84	−2.10	−1.23	−3.52	1.523
Δ ECM yield	−2.47	−6.81	−2.85	−6.24	2.153
Δ Fat yield	−0.14	−0.39	−0.19	−0.34	0.142
Δ Protein yield	−0.09	−0.19	−0.05	−0.17	0.075
Δ Fat concentration	−3.38	−8.39	−4.37	−5.88	3.286
Δ Protein concentration	−1.71 ^b^	−4.06 ^a^	−0.18 ^b^	−1.88 ^ab^	1.003

^1^ BLY = basal diet plus barley grain; CAN = basal diet plus canola meal and wheat grain; CRN = basal diet plus corn grain; WHT = basal diet plus wheat grain. ^2^ ECM = energy-corrected milk. ^3^ Δ variable = (heat-challenge variable − pre-challenge variable). ^a–c^ Means in the same row followed by different superscripts differ significantly (*p <* 0.05).

**Table 5:** Vaginal temperature (°C) and duration > 38.8 °C (mins), respiration rate (breaths per minute) and skin temperature (neck, °C) during the periods of the experiment.

	BLY ^1^	CAN	CRN	WHT	SED
*Animals (n)*	5	3	6	4	
*5-day mean*					
Mean	38.4 ^a^	38.9 ^b^	38.9 ^b^	38.7 ^ab^	0.20
Minimum	37.74 ^a^	38.07 ^ab^	38.14 ^b^	37.91 ^ab^	0.142
Maximum	39.3 ^a^	40.0 ^b^	39.9 ^b^	39.5 ^ab^	0.29
Duration > 38.8 °C	318 ^a^	675 ^b^	625 ^b^	433 ^ab^	141
Respiration	65.6	78.3	65.2	70.2	12.70
Skin temperature	33.5 ^a^	35.6 ^b^	35.2 ^b^	34.3 ^ab^	0.75
*Pre-challenge*					
Mean	38.2	38.6	38.7	38.4	0.24
Minimum	37.8 ^a^	38.0 ^ab^	38.2 ^b^	37.8 ^a^	0.19
Maximum	38.7	39.2	39.2	38.9	0.34
Duration > 38.8 °C	12	404	251	113	191
Respiration	39.2	45.2	42.4	43.9	14.50
Skin temperature	31.2 ^a^	33.0 ^ab^	34.2 ^b^	32.5 ^ab^	1.43
*Heat challenge*					
Mean	38.9 ^a^	39.3 ^ab^	39.5 ^b^	39.2 ^ab^	0.22
Minimum	38.1 ^a^	38.3 ^ab^	38.5 ^b^	38.3 ^ab^	0.16
Maximum	39.7 ^a^	40.5 ^b^	40.4 ^b^	39.9 ^ab^	0.31
Duration > 38.8 °C	777 ^a^	1068 ^ab^	1157 ^b^	934 ^ab^	160
Respiration	93.8	97.1	91.0	101	13.48
Skin temperature	35.6	35.8	36.7	35.6	1.03
*Recovery*					
Mean	38.0 ^a^	38.7 ^b^	38.5 ^ab^	38.3 ^a^	0.22
Minimum	37.4 ^a^	38.0 ^b^	37.7 ^ab^	37.6 ^a^	0.17
Maximum	39.2 ^a^	40.0 ^b^	39.7 ^ab^	39.3 ^a^	0.31
Duration > 38.8 °C	14 ^a^	417 ^b^	281 ^ab^	93 ^ab^	161
Respiration	50.7	76.0	50.8	52.9	13.45
Skin temperature	32.5 ^a^	36.6 ^b^	34.3 ^ab^	33.9 ^ab^	1.41
*Before challenge with heat * ^2^					
Δ Mean	0.70	0.67	0.81	0.80	0.165
Δ Minimum	0.28	0.25	0.27	0.45	0.158
Δ Maximum	1.04	1.26	1.15	1.01	0.251
Δ Duration > 38.8 °C	764	664	906	820	182
Δ Respiration	54.6	51.8	48.6	56.8	9.63
Δ Skin temperature	4.4	2.8	2.4	3.1	1.76

^1^ BLY = basal diet plus barley grain; CAN = basal diet plus canola meal and wheat grain; CRN = basal diet plus corn grain; WHT = basal diet plus wheat grain. ^2^ Δ variable = (heat-challenge variable − pre-challenge variable). ^a,b^ Means in the same row followed by different superscripts differ significantly (*p* < 0.05).

**Table 6:** Selected blood parameters during selected periods of the experiment. Blood pH and serum concentrations of beta-hydroxy butyrate (BHB; mmol/L), non-esterified fatty acids (NEFA; mmol/L), glucose (mmol/L), haptoglobin (g/L), Na+ (mmol/L) and K+ (mmol/L).

	BLY ^1^	CAN	CRN	WHT	SED
*Animals (n)*	5	3	6	4	
*Pre-challenge*					
pH	7.11	7.13	7.18	7.15	0.055
BHB	0.97	0.60	0.93	0.53	0.257
NEFA	0.17	0.13	0.11	0.08	0.061
Glucose	3.08	2.98	3.02	3.39	0.287
Haptoglobin	0.25 ^b^	0.03 ^a^	0.17 ^ab^	0.13 ^ab^	0.097
Na	138 ^ab^	139 ^ab^	135 ^a^	140 ^b^	2.2
*Heat challenge*					
pH	7.15	7.12	7.14	7.14	0.054
BHB	0.60	0.71	0.63	0.44	0.257
NEFA	0.32 ^b^	0.46 ^c^	0.19 ^a^	0.17 ^a^	0.061
Glucose	3.26	3.13	3.43	3.41	0.287
Haptoglobin	0.17	0.03	0.16	0.12	0.097
Na	137	135	135	133	2.1
*Recovery*					
pH	7.23	7.16	7.21	7.26	0.061
BHB	0.77	0.73	0.80	0.42	0.253
NEFA	0.09	0.16	0.15	0.06	0.060
Glucose	2.93	2.90	2.87	3.07	0.283
Haptoglobin	0.03	0.11	0.16	0.06	0.095
Na	133	135	134	134	2.4
*Before challenge with heat* ^2^					
Δ pH	0.04	−0.01	−0.05	−0.01	0.076
Δ BHB	−0.37	−0.11	−0.30	−0.08	0.274
Δ NEFA	0.15 ^a^	0.33 ^b^	0.08 ^a^	0.09 ^a^	0.078
Δ Glucose	0.18	0.16	0.40	0.01	0.336
Δ Haptoglobin	−0.08	0.00	−0.02	−0.01	0.116
Δ Na	−1.4 ^ab^	−4.2 ^ab^	0.1 ^b^	−6.4 ^a^	2.98

^1^ BLY = basal diet plus barley grain; CAN = basal diet plus canola meal and wheat grain; CRN = basal diet plus corn grain; WHT = basal diet plus wheat grain. ^2^ Δ variable = (heat-challenge variable − pre-challenge variable). ^a–c^ Means in the same row followed by different superscripts differ significantly (*p* < 0.05).

## Data Availability

Data are available from the corresponding author upon reasonable request.

## Supplementary Materials

The following supporting information can be downloaded at https://www.mdpi.com/article/10.3390/ani15071045/s1, Table S1. Composition of individual diet ingredients (g/kg DM unless otherwise stated) used in this experiment. Table S2. Blood parameters during selected periods of the experiment.
